# New Appliances and Things Medical

**Published:** 1894-07-28

**Authors:** 


### NEW APPLIANCES AND THINGS MEDICAL.

[All preparations, appliances, novelties, &c., of which a notice is desired, should be sent for the Editor, to care of The Manager, 428, Strand, London, W.C.]

### DR. RIDGE'S PATENT COOKED FOOD.

Samples of this well-known brand have been forwarded for our approval. In the nursery and sick-room it has long been a valued friend, but in the preparation of custards, baked pudding, and as a farinaceous addition to beef tea, broth or soup, it should be remembered as a valuable household possession. It is composed of a judicious admixture of selected flours, containing about 1·13 per cent. of salts, about 2 per cent. of fats, about 10 per cent. of albuminoids, and about 82 per cent. of carbo-hydrates. It will, therefore, be recognised that as an addition to milk, especially such as is rich in cream, it is calculated to form an almost perfect physiological food, provided that it is not used for infants under six months of age, who are incapable of digesting farinaceous foods.

